# Demonstration of a diamond anvil cell platform at the Linac Coherent Light Source: capabilities and outlook

**DOI:** 10.1107/S1600577526001608

**Published:** 2026-03-26

**Authors:** Mungo Frost, Nina Boiadjieva, Minkyung Han, Quynh L. Nguyen, Mengnan Wang, Hannah Bartels, Eric Galtier, Shao Xian Lee, Hemamala I. Karunadasa, Gilliss Dyer, Siegfried H. Glenzer, Wendy L. Mao, Yu Lin, Hae Ja Lee

**Affiliations:** a SLAC National Accelerator Laboratory 2575 Sand Hill Road Menlo Park CA94025 USA; b Department of Earth and Planetary Sciences Stanford University Stanford CA94305 USA; c Department of Chemistry Stanford University Stanford CA94305 USA; RIKEN SPring-8 Center, Japan

**Keywords:** diamond anvil cell (DAC), Linac Coherent Light Source (LCLS), X-ray free electron laser (XFEL), diffraction, high pressure

## Abstract

A novel X-ray free electron laser (XFEL) diffraction setup for use with diamond anvil cells (DACs) at the Linac Coherent Light Source (LCLS) is described. The new diamond window at the Matter at Extreme Conditions (MEC) instrument allows hard X-ray experiments on DACs to be performed in air. The platform is described along with alignment and calibration procedures, and details of the X-ray beam and diagnostics. Example data are presented, including a reversible XFEL-induced phase transition in CsPbI_3_. The DAC setup was commissioned at MEC, but is applicable to most LCLS instruments where the unique pulse structures available at LCLS offer access to new ultrafast experimental techniques at high pressure.

## Abstract 2

A setup for fielding high-pressure samples contained in diamond anvil cells (DACs) at the LCLS X-ray free electron laser is described and example powder X-ray diffraction data are presented. Future prospects for experimental capabilities in DACs enabled by the LCLS, such as short pulse laser interactions and multiple pulse modes, are discussed.

## 1. Introduction

The development of X-ray free electron laser (XFEL) radiation sources offers X-rays of unprecedented brilliance and enables a wide range of novel experiments, particularly in the investigation of transient phenomena and in time-resolved measurements (Seiboth *et al.*, 2018 ▸; Husband *et al.*, 2024 ▸). The capability of producing hard X-rays, with photon energies greater than 13 keV, has opened the path to using XFEL sources on high-pressure samples compressed in diamond anvil cells (DACs) (Liermann *et al.*, 2021 ▸). Samples in DACs are statically compressed between diamond anvils and contained within a metallic gasket. Diamond is transparent to a wide range of probes, including hard X-rays. A typical diamond anvil is around 1.5 mm thick, with a total path length of 3 mm through both anvils, which matches the attenuation length of 13300 eV X-rays in diamond. Thus, ∼13 keV serves as a lower practical energy for DAC experiments. Harder X-rays are desirable in many cases due to the limited angular access in standard DAC designs. Due to the necessity of supporting materials and seats for the anvils, angular access to the sample is limited to a cone of 60 to 90° included angle. Going to higher photon energy, and shorter wavelength, allows higher *q* phenomena to be probed within the restrictions of the DAC.

Given these limitations, DAC experiments at XFEL sources have previously been conducted only at PAL-XFEL (Pace *et al.*, 2020 ▸) and the European XFEL (EuXFEL) (Liermann *et al.*, 2021 ▸). Nonetheless, many studies have found the diamond anvils to be robust to the intense XFEL pulses and these have enabled several new regimes of experiment. X-ray pump–probe experiments, which use sequential XFEL pulses as both a pump to heat the sample and a probe to interrogate the result of the previous pulse, have enabled a range of high-temperature studies looking at chemical reactions (Frost *et al.*, 2025 ▸), phase transitions (Husband *et al.*, 2024 ▸), and thermal conductivity (Edmund *et al.*, 2024 ▸), among many others. Pulsed laser heating coupled with XFEL pulses is an effective alternative to the X-ray pump–probe, where a nanosecond laser pulse transiently heats the sample and the XFEL probes the heated state before and during cooling (Jaisle *et al.*, 2023 ▸). Limiting the duration at high temperature improves the survivability of the diamond anvils and allows access to higher temperature than conventional continuous wave laser heating and can reduce sample contamination and chemical migration.

Direct modifications of material properties and chemistry *via* the promotion of substantial populations of core electrons by the extremely intense X-rays have also been reported (Siska *et al.*, 2024 ▸; Konopkova *et al.*, 2025 ▸). Another thrust is in the field of dynamic DACs, where extreme strain rates can be generated using piezoelectric actuators (Husband *et al.*, 2023 ▸). The extremely short pulse durations of XFEL sources, typically <50 fs, are ideal to probe material responses to the rapid change in conditions.

Despite the significant efforts made developing DAC methods on XFEL sources in recent years, there are still unanswered questions about the response of the samples on short timescales from picoseconds to a few nanoseconds after irradiation by an XFEL pulse. The energy is deposited primarily into the electrons during the <50 fs XFEL pulse, which is much faster than the timescale of nuclear motion. In this, there is promotion of core electrons (Alonso-Mori *et al.*, 2020 ▸), creation of nonthermal states (Beyerlein *et al.*, 2018 ▸), and, according to finite element models, transient extremely high temperatures (Meza-Galvez *et al.*, 2020 ▸). In the first few picoseconds, electron–phonon coupling will equilibrate the electronic temperature with that of the ions (Mo *et al.*, 2024 ▸), resulting in nearly isochoric heating. Where the sample is higher *Z*, and so more strongly absorbing, than the diamond anvils or low-*Z* insulation (Meza-Galvez *et al.*, 2020 ▸), the sample will be considerably hotter than the surrounding material after electron–ion equilibration. Thermal pressure will then cause expansion of this material into the cooler material around it. This expansion will drive intense com­pres­sion waves, or potentially shocks, into the insulating material. These com­pres­sion waves will be partially reflected back at the sample to the diamond anvil interface and converge, resulting in complex reverberations.

The highest repetition rate used in DACs is 4.5 MHz at EuXFEL, with 222 ns between pulses. This is long enough for the complex states immediately after heating to relax (Meza-Galvez *et al.*, 2020 ▸). Differences observed between conventional laser heating and XFEL DAC studies (Weck *et al.*, 2022 ▸; Husband *et al.*, 2024 ▸), and between XFEL DACs and dynamic com­pres­sion (Singh *et al.*, 2023 ▸; Konopkova *et al.*, 2025 ▸), may arise from these transient states shortly after the XFEL pulse–sample interaction, motivating their study.

The Linac Coherent Light Source (LCLS) offers unique capabilities in terms of X-ray pulse structures, which can enable a range of experiments probing short-time phenomena. Some current capabilities are outlined in Table 1 ▸. By tightly co-timing pairs or series of pulses, the state of samples contained within a DAC immediately after interaction with an XFEL pulse can be probed. Additionally, other ultrafast phenomena, such as electron–phonon coupling, laser-driven transitions, and transient states, such as warm dense matter induced either directly by X-rays or by interaction with short pulse lasers, are accessible. This motivates bringing together the capabilities of LCLS and static DAC com­pres­sion of samples in a manner complimentary to existing facilities.

## 2. Experimental setup

For this proof-of-concept commissioning experiment, the LCLS operated in standard single pulse mode using the 120 Hz hard X-ray copper linac. The photon energy was 16.6 keV with a typical pulse energy of 0.6 mJ, which was attenuated to 40% by the transmission of the beamline optics at MEC. Absorbers were used to further attenuate the beam in the range of 1 to 100% of that available (2.4 to 240 µJ). The beam was focused using Be compound refractive lenses (CRLs) to a spot size of 10 µm full width at half-maximum (FWHM). The X-ray parameters used are outlined in Table 2 ▸. Table 3 ▸ outlines multi-pulse structures which are relevant to DAC studies and available at LCLS, but which were not used in this commissioning experiment.

Due to the high photon energy generally used in X-ray DAC experiments, there is no need to operate under vacuum, and so the experiment was carried out in air using the newly commissioned diamond window in the MEC target chamber. The in-air X-ray path from the diamond window to the detectors was approximately 1.5 m, resulting in around ∼20% attenuation by the air. Given the increased experimental efficiency of not needing to pump the chamber when changing samples, this trade-off is advantageous for most experiments. It should be noted that air scatter will contribute a broad background. While this can usually be subtracted along with the diffuse scatter from the diamond anvils, the setup described here could also be run in a vacuum if required.

The diamond window consists of a polycrystalline diamond wafer which is 100 µm thick and 10 mm in diameter, such that at the 16.6 keV used it transmits more than 98% of the in­coming beam. Other than very slight attenuation it has negligible effect on the XFEL beam characteristics. As well as facilitating rapid sample exchange by obviating the need to pump down, it simplifies DAC preparation as vacuum incompatible materials may be used. It also opens the option for online membrane pressure control, such as is used in other X-ray facilities (Bommannavar *et al.*, 2022 ▸), which operate in air, but which are difficult to adequately seal for use under high vacuum.

**Figure 1:**
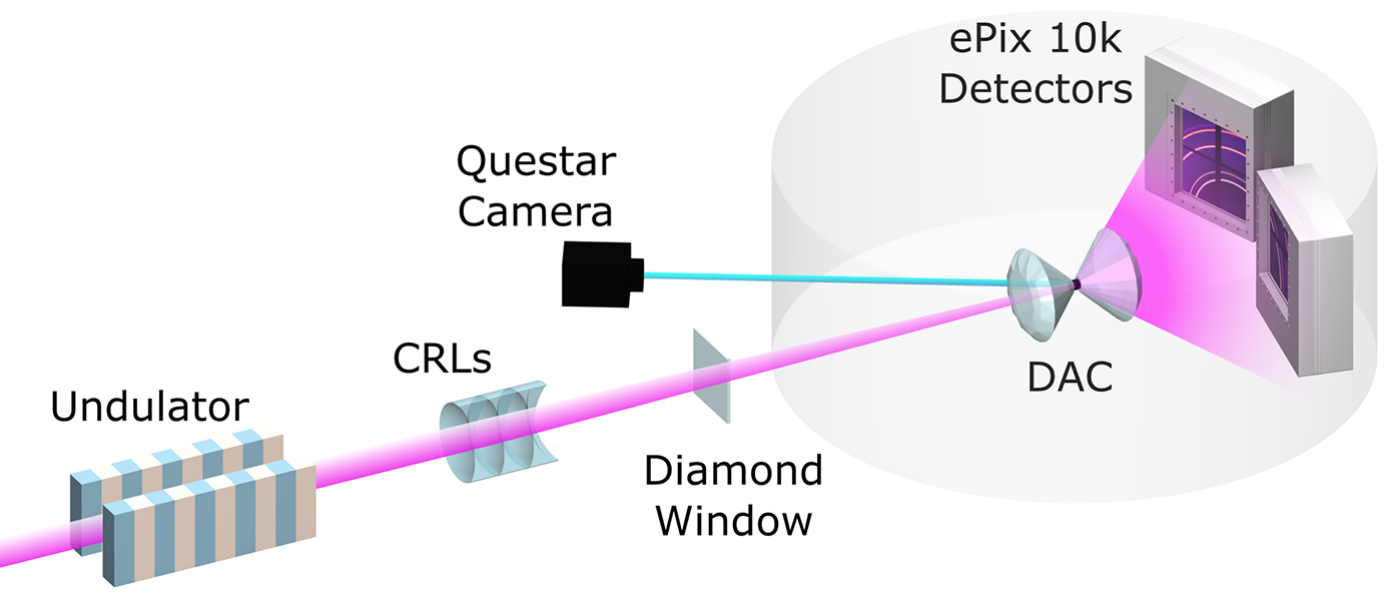
Diagram of the experimental setup in the MEC target chamber. The 16.6 keV XFEL beam is shown in pink and the viewing axis of the Questar in blue. Upstream (left) of the diamond window is in a vacuum and the other components are in air. The transparent gray ring indicates the MEC chamber.

A diagram of the MEC chamber layout used is shown in Fig. 1 ▸. The incident XFEL pulses are cleaned and focused as described elsewhere (Nagler *et al.*, 2015 ▸), which includes gas monitors to measure the intensity of the XFEL pulses and beryllium CRLs to focus the beam. The DACs were mounted on a stage stack consisting of a hexapod six-axis positioner and linear stage for longer range movement transverse to the beam. This was positioned in the MEC chamber ∼1300 mm downstream from the diamond window. Wide-angle X-ray diffraction data were collected on two ePix10k quad detectors, though, in principle, up to four quads may be routinely fielded at MEC. The ePix10k quad is a 2D pixel array detector utilizing 100 µm thick silicon over a 80 mm × 80 mm sensing area with a 100 µm pixel size. The ePix10k quad operates at the 120 Hz rate of the XFEL individually resolving each pulse with high dynamic range from single photon counting to 10000 photons/pixel/pulse at 8 keV. For a full description, refer to Van Driel *et al.* (2020 ▸).

The detectors are easily moved to cover different *q* ranges, and two positions were investigated during the commissioning beamtime. The MEC chamber is 2 m in diameter and the sample stage can be positioned at any point along the XFEL beam, which goes centrally through it. Likewise, the detectors can be freely positioned in the chamber, provided the direct XFEL beam does not impinge on them, allowing coverage at any angle. In DAC experiments, the geometry of the cell, seats, and diamond anvils usually restrict diffraction to, at most, a 90° included angle cone downstream of the sample, while limited sample volume and non-ideal powders make azimuthal coverage important. This can be optimized by clustering the detectors behind the sample at a distance such that the required *q* range is covered by their 80 mm × 80 mm sensing areas. The Δ*E*/*E* of the self-amplified spontaneous emission (SASE) beam is ∼0.2%, which will be the dominant limitation on *q* resolution where it is used.

**Figure 2:**
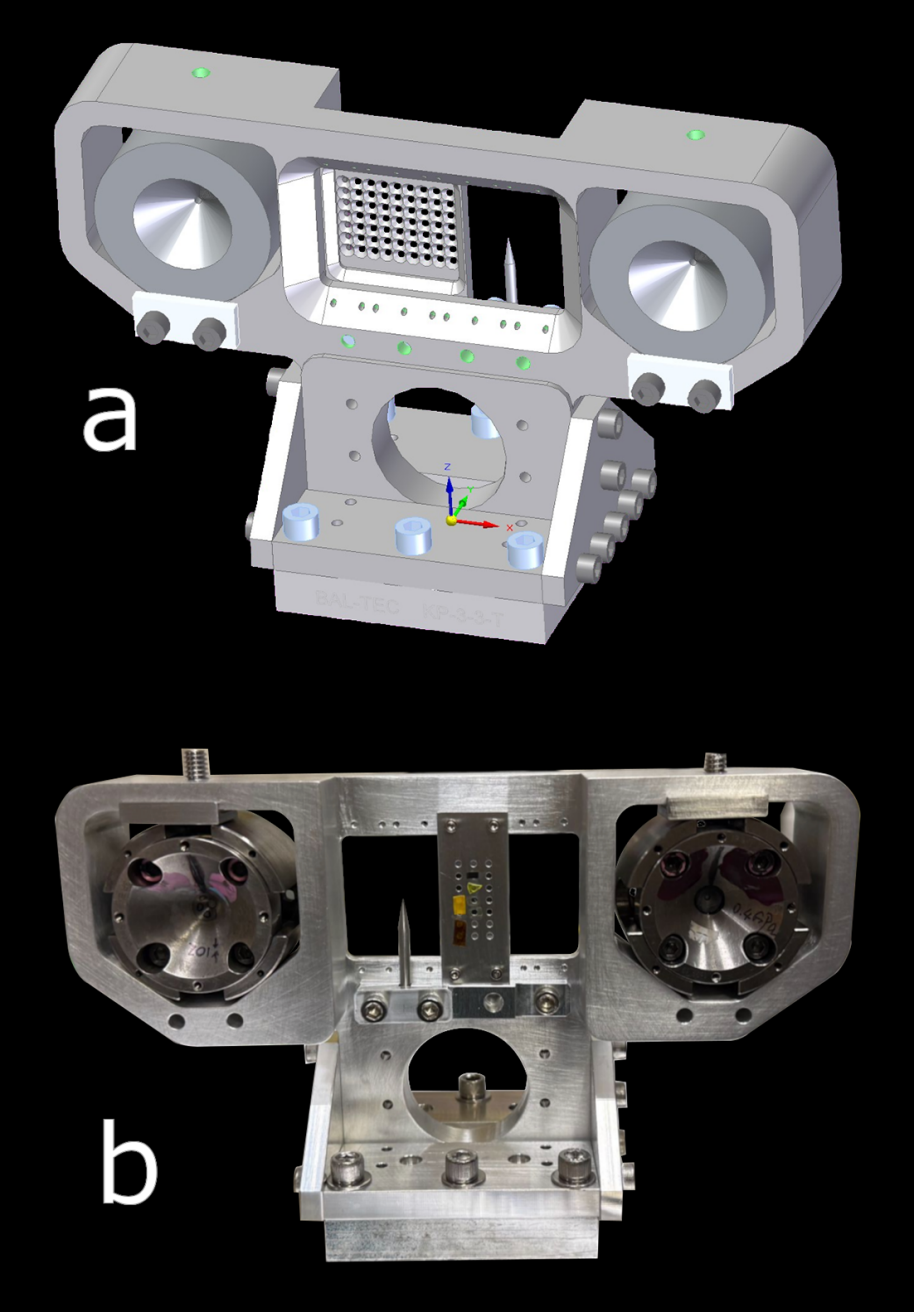
(*a*) A render of the assembled DAC mount with two DACs installed either side of the alignment card and pin, viewed from the downstream side. The tip of the pin is at the same nominal height and position along the beam axis as the DAC samples. (*b*) A photo of the holder in use, viewed from the upstream side. The DACs are BX90 type with a 50 mm diameter.

The DACs were mounted on a custom-designed holder shown in Fig. 2 ▸. This holds two DACs, so both can be measured without breaking the hutch interlock, along with a standard MEC sample card, which carries calibration and alignment diagnostics, and a pin, which is positioned at the same height and position along the beam axis as the DAC samples. The holder as designed is optimized for BX90-type DACs (Kantor *et al.*, 2012 ▸), though any cell with a diameter equal or less than 50.4 mm (2 inch) can be used, so long as the vertical and beam-axis offsets from the pin position are noted for the purposes of rough alignment.

The mount is made from aluminium and supports the DACs on Vs with centers spaced at 140 mm symmetrical about the holder center. Back plates provide stops which constrain the DACs on the beam axis, and they are held in place from the top with set screws which engage pusher plates. It has a kinematic mount on the base for fast and repeatable removal and replacement on the chamber stage.

### 2.1. DAC alignment and calibration

The DAC holder includes a space for a calibration card, which allows the calibrants and alignment aids to be tailored to the requirements of each sample. It is positioned at the same nominal position on the axis of the beam as the samples in the BX90-type DACs. Small offsets arising from variations in the thickness of the diamond anvils, seats, and cell are cor­rected during the alignment procedure. For this experiment, it held both CeO_2_ and LaB_6_ powders to calibrate the detectors, and a cerium-doped yttrium aluminium garnet (Ce:YAG) screen, the fluorescence of which allows imaging of the XFEL beam with the Questar long-distance alignment microscope (Glenzer *et al.*, 2016 ▸). The YAG screen lies on the same plane as the sample calibrants.

The Questar camera offers a large field of view but less detail compared to the online imaging at most other X-ray facilities optimized for DAC studies. It also has a large depth of field which makes it unsuitable for setting the sample-to-detector distance using its focal plane. However, with the aid of a fragment of YAG glued to the table of the upstream anvil, precise alignment of DACs on the XFEL is possible using the Questar imaging system. The camera is positioned above the XFEL beam and upstream from the sample such that it images at a 15° angle to the beam. This can be used to set the sample distance.

**Figure 3:**
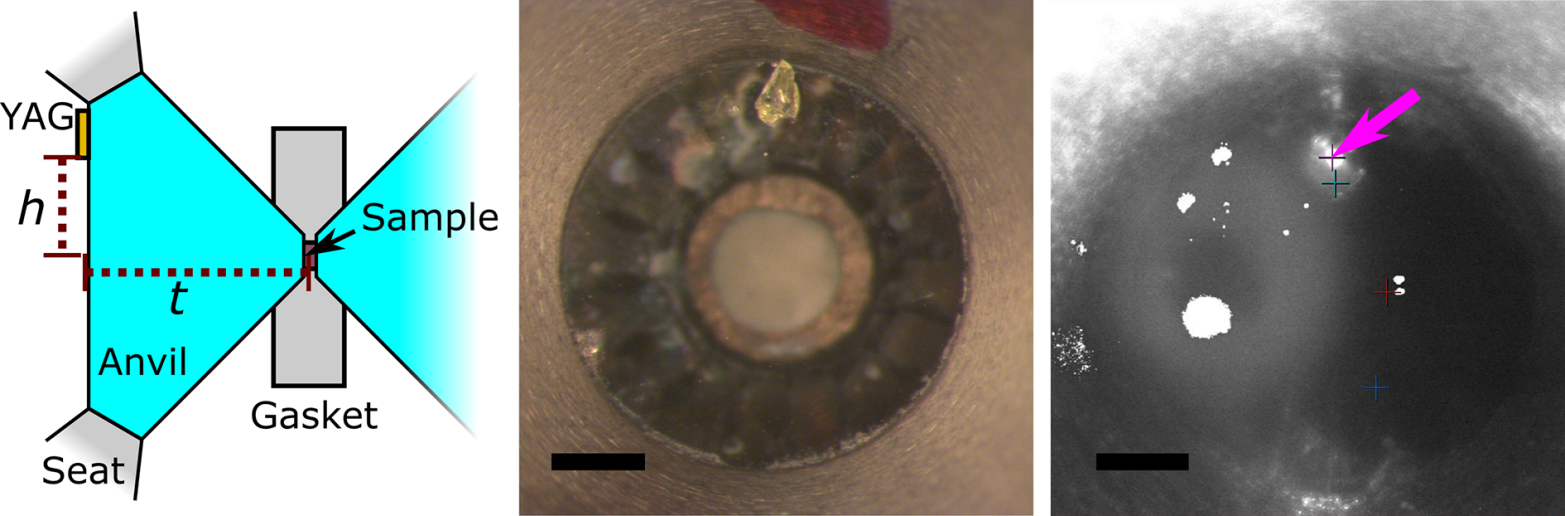
A YAG fragment is glued to the table of the upstream anvil to allow for alignment. (Left) A diagram indicating the anvil thickness, *t* and offset from YAG to sample, *h* which are measured in advance. (Middle) Photograph of table with adhered YAG. The CeO_2_ sample visible through the anvil is shifted slightly to the right by microscope parallax. (Right) Image of XFEL beam visible on YAG fragment during alignment (fuchsia arrow). The halo to the left of the image is a reflection of the light, other white sections are damaged pixels. Scale bars are 200 µm.

The thickness of the upstream anvil is measured prior to sample preparation and a small fragment of Ce:YAG is glued to its table (the outer air-facing side of the anvil). An image of a prepared cell is shown in Fig. 3 ▸. The offset from the edge of the YAG to the sample is measured, then the DAC is mounted such that the YAG fragment is vertically above the sample in the holder.

**Figure 4:**
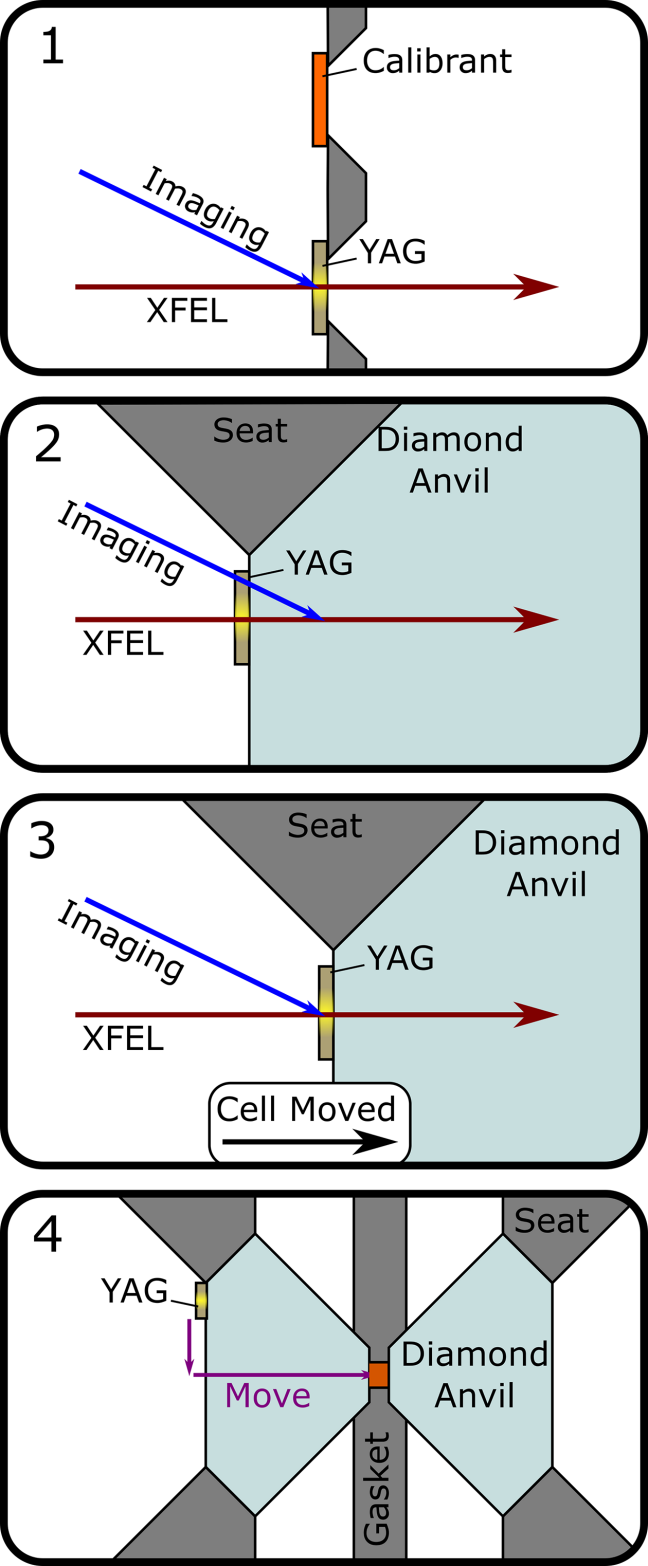
Diagram of the alignment procedure. (**1**) The position of the XFEL beam on the imaging camera at the calibration plane is noted using the YAG screen on the calibration card. (**2**) The back of the upstream anvil is imaged and positioned such that the XFEL hits the YAG. (**3**) The cell is translated on the beam axis until the image of the XFEL spot is at the same location as it was on the calibration YAG screen in panel **1**. (**4**) The cell is moved by the previously measured offset to get from the YAG on the upstream anvil to the sample.

The sample is aligned as illustrated in Fig. 4 ▸. After calibration of the detector, the position of the XFEL beam on the YAG screen imaged with the Questar camera is noted. The stage is then moved based on the known geometry of the holder to image the table of the upstream anvil. The highly attenuated XFEL beam is then turned on and found on the YAG fragment on the DAC table (Fig. 3 ▸, right). Due to the angle between the XFEL beam and the Questar imaging system, moving the DAC along the axis of the beam causes the image to move vertically, and when the XFEL spot is in the same position on the image as it was on the YAG screen during calibration, the YAG on the DAC is at the calibration plane (Fig. 4 ▸, middle panels). The cell can then be translated to account for the, previously measured, thickness of the upstream anvil and YAG fragment, and vertically for the measured offset from the YAG fragment to the sample. The Questar camera has 20 µm resolution which, combined with the 15° angle between the imaging axis and the beam, allows alignment along the beam to better than 80 µm and about 20 µm precision perpendicular to the beam. Uncertainties arising from the measurement of the anvil thickness will generally be much less than 80 µm, so the imaging is the dominant source of uncertainty in setting the sample–detector distance.

This method proved successful with all DACs, the smallest culets being 400 µm. It is different from the method used at the EuXFEL High Energy Density (HED) instrument, and has advantages and drawbacks. At the EuXFEL HED, an image of the sample is found on a higher resolution microscope using the focal plane to position the sample plane on the beam axis. Then the gasket is briefly exposed to the XFEL beam at sufficient power to leave a mark, referred to as ‘imprinting’ the gasket. This gives precise detail of the position of the beam on the image so the cell can then be translated to align the beam with specific features or very small samples. However, the imprinting process can also cause damage to the anvils and in some cases cell failure and does not work in all cases. Due to the refractive index of the anvil, its thickness must still be known with this alignment method so it can be accounted for in the detector calibration.

The new method using a YAG fragment used here avoids exposing the cells to intense radiation during alignment, and so avoids potential damage to the gasket, anvils, and sample. However, in its current state, it does not offer as precise an alignment with individual sample features. For example, the perforated foil couplers made from high-*Z* foils with hole diameters similar to that of the XFEL beam that have been used for the XFEL heating of lower-*Z* samples (Frost *et al.*, 2024 ▸; Husband *et al.*, 2024 ▸) would be impractical to align with the current procedure. With improved imaging it is likely that this could be overcome and the method presented here may be applicable elsewhere.

## 3. Example data

Four DACs were investigated in the first commissioning experiment. These are outlined in Table 4 ▸ and consisted of CeO_2_ at 4 GPa, chromium powder with ammonia borane (NH_3_BH_3_) at 26 GPa, and two powder samples of CsPbI_3_ at 0.4 and 1.2 GPa. These are discussed in turn with example data presented.

### 3.1. Cerium dioxide

Cerium dioxide has been widely studied under high pressure (Liu *et al.*, 2012 ▸; Frost *et al.*, 2021 ▸) and is commonly used as a calibrant in X-ray diffraction experiments. The wide familiarity with the compound makes it ideal to illustrate the quality of powder diffraction data obtainable from DACs at LCLS. CeO_2_ undergoes a phase transition from an *Fm*
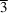
*m* fluorite structure to a *Pnam* α-PbCl_2_ structure starting at about 30 GPa (Liu *et al.*, 2012 ▸). Since the pressure studied here was lower, the sample remained in the *Fm*
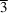
*m* phase.

Cerium dioxide (Acros Organics, 99.9% trace metals basis) was loaded into a DAC equipped with anvils with 1000 µm culets and a stainless steel gasket preindented to 102 µm with a 750 µm hole cut using electrical discharge machining. Both anvils were of the Boehler–Almax design (Boehler & De Hantsetters, 2004 ▸), the upstream anvil was 1.54 mm thick, and the downstream anvil was 1.27 mm, as measured using a micrometer. Before fielding at the LCLS, the sample was compressed to 4 GPa using a ruby pressure marker at one edge (Shen *et al.*, 2020 ▸). To avoid potential reactions, it was loaded without a pressure-transmitting medium or the low-*Z* insulation generally used to protect the diamond anvils in XFEL DAC experiments (Liermann *et al.*, 2021 ▸; Meza-Galvez *et al.*, 2020 ▸). The CsPbI_3_ samples (see Section 3.3), also omitted insulators. In all cases, the diamond anvils were undamaged. This may be due to the low pressures, or may indicate that the anvil damage mechanism from XFEL interactions with high-*Z* samples in DACs is more complex than previously thought and likely also involves the sustained high temperatures occurring in MHz pulse trains at EuXFEL.

**Figure 5:**
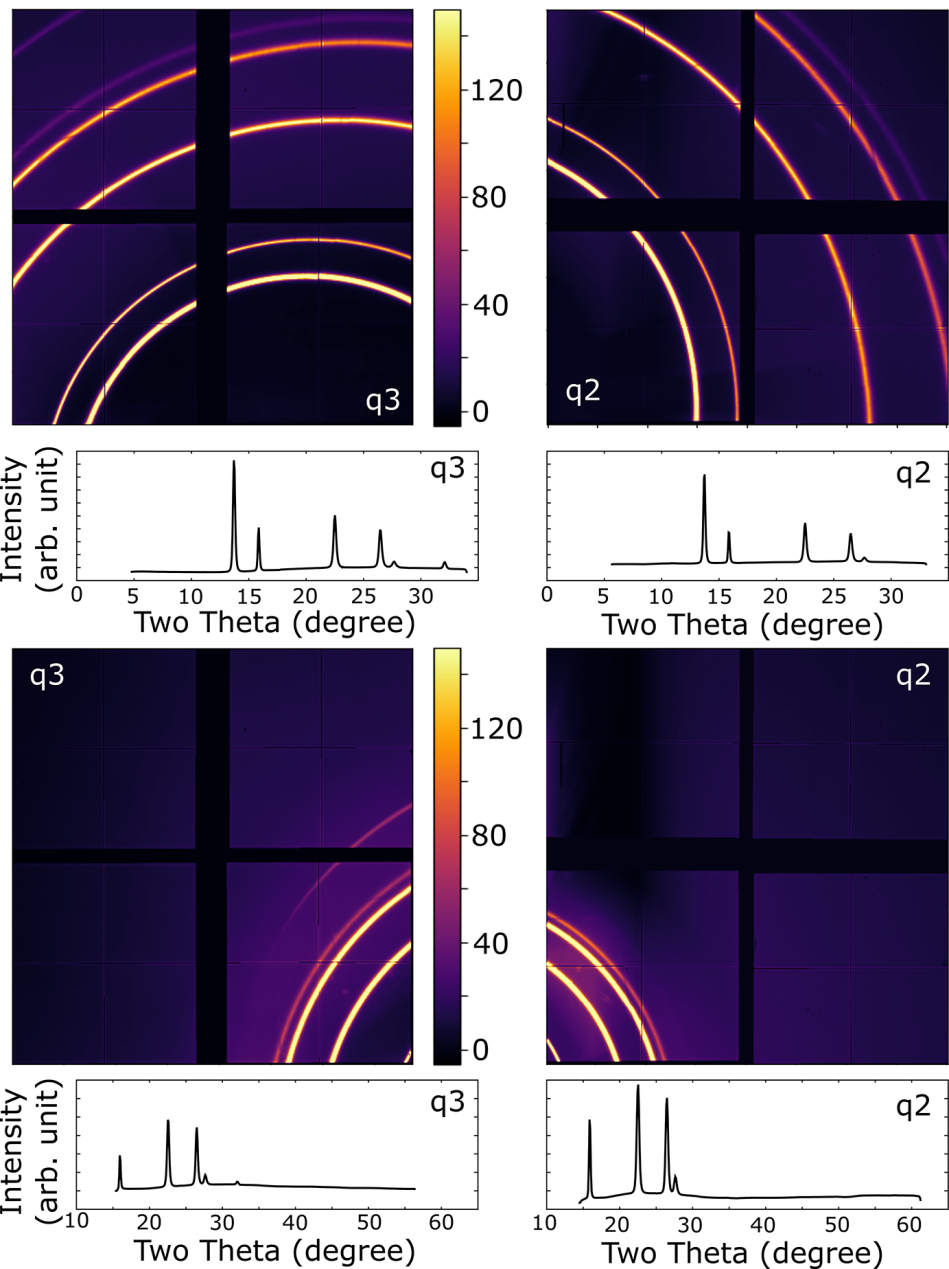
X-ray diffraction of CeO_2_ at 4 GPa taken at 25% beam intensity showing images and integrated patterns. (Top) Mean of the diffraction patterns from 2705 XFEL pulses, with detectors ‘q2’ and ‘q3’ at 197 and 167 mm from the sample, respectively. These positions were used for the other samples. (Bottom) Mean of the diffraction patterns from 894 pulses, with detectors ‘q2’ and ‘q3’ at 84 and 113 mm from the sample, respectively. This provides an angular coverage beyond what is accessible in most DAC designs. The color scale is the same for all patterns.

Fig. 5 ▸ shows data collected from this sample. As can be seen, the data quality is excellent. Two detector positions are shown and the detectors are easily moved as required for the experiment. No changes in the sample were observed on repeated exposure to XFEL pulses, with the highest pulse energy run being 77 (20) µJ at 40% transmission.

### 3.2. Chromium in ammonia borane

Ammonia borane is often used as a solid hydrogen source for high-pressure experiments. On heating under pressure, it decomposes into molecular H_2_ and boron nitride. The boron nitride is unreactive and typically does not take part in subsequent reactions (Antonov *et al.*, 2017 ▸). Chromium metal has a body-centered cubic structure and is known to form hydrides under pressure. Above 3 GPa it will form a hexagonal *P*6_3_/*mmc* monohydride, which converts to Cr_2_H_3_ with *C*2/*m* symmetry above 19 GPa (Marizy *et al.*, 2018 ▸).

Chromium (Thermo Scientific, 99% metals basis) and ammonia borane, NH_3_BH_3_ (Sigma–Aldrich, 95%), were mixed by grinding in an agate mortar prior to being loaded into a DAC with Boehler–Almax anvils with 400 µm culets and a rhenium gasket preindented to 51 µm thickness with a 220 µm laser-cut sample hole (Frost *et al.*, 2020 ▸). The sample was then compressed to 26 GPa prior to being investigated at the LCLS.

**Figure 6:**
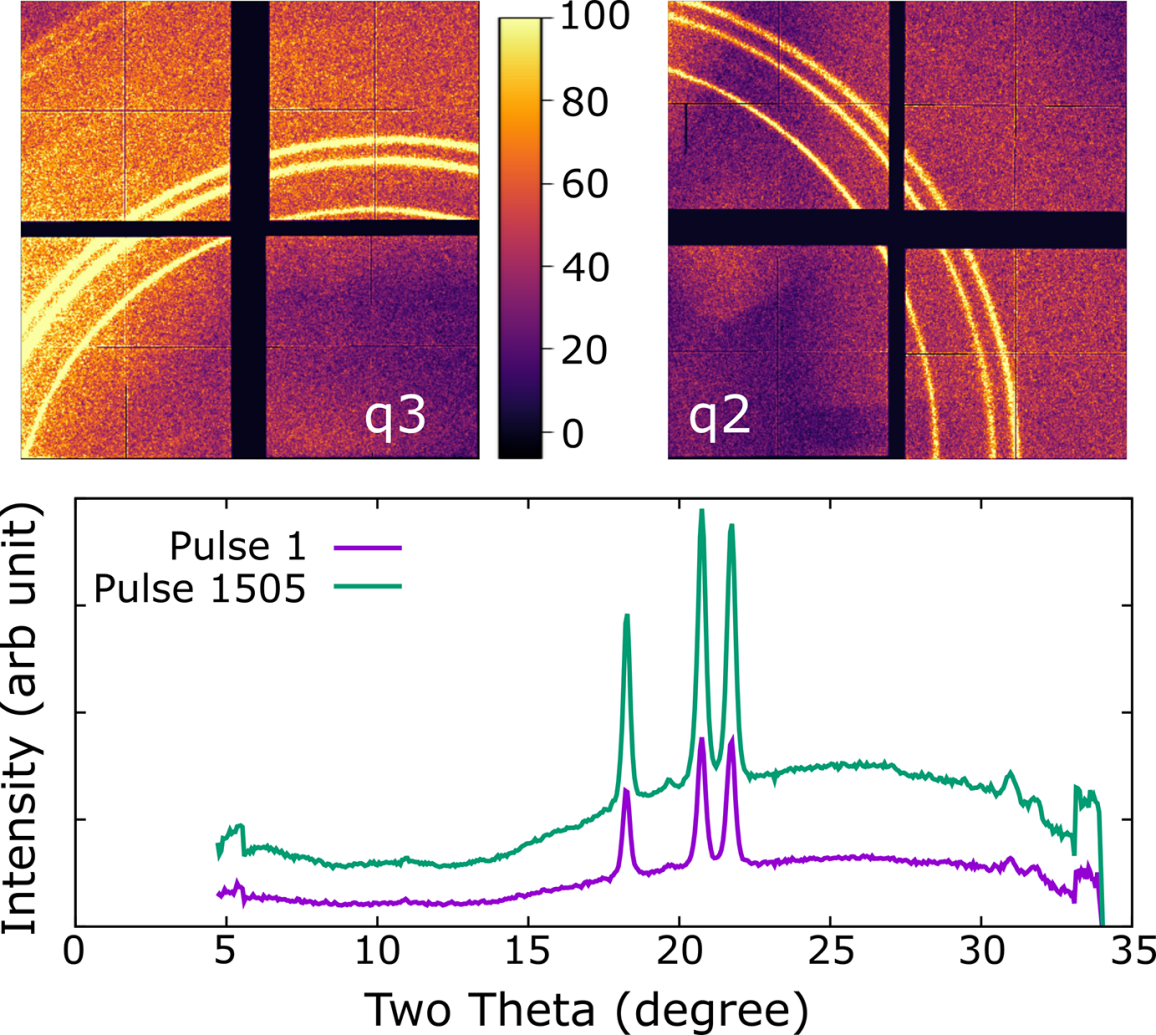
Diffraction from chromium in ammonia borane. (Top) Diffraction images from an individual XFEL pulse (1505th on sample) for each detector. The color scale is the same for both images. (Bottom) Integrated diffraction patterns from both images combined for the first and 1505th pulses of a run. No change is observed. The run had a pulse energy of 99 (27) µJ (50% transmission).

Diffraction results are shown in Fig. 6 ▸. At the start, the sample was observed to have partially reacted to form a hydride phase. The peaks are not compatible with *P*6_3_/*mmc* CrH, but appear similar to those indexed as *C*2/*m* Cr_2_H_3_ in a previous study (Marizy *et al.*, 2018 ▸). X-ray transmissions of up to 100%, corresponding to measured pulse energies of 206 (48) µJ, were used with 1034 pulses incident on the sample at highest fluence. No further reaction was observed. The formation of Cr_2_H_3_ prior to heating implies that chromium can dehydrogenate ammonia borane at high pressure and ambient temperature.

### 3.3. CsPbI_3_

CsPbI_3_ is an attractive candidate for XFEL studies because its heavy-element composition enables efficient X-ray heating and X-ray diffraction. At high temperatures, CsPbI_3_ undergoes a series of phase transitions from the non-per­ov­skite δ-phase to the cubic per­ov­skite α-phase, which upon cooling transforms to the tetragonal β- and orthorhombic γ-phases through inter-octahedral tilting (Marronnier *et al.*, 2018 ▸). Phase (meta)stability can be further manipulated by external pressure (Ke *et al.*, 2021 ▸) or strain (Straus *et al.*, 2019 ▸). By fine-tuning the applied pressure or the strain level, as well as the heating and cooling rates, the metastable per­ov­skite phases can be preserved to ambient conditions for extended periods. Two δ-phase CsPbI_3_ samples were loaded without a pressure-transmitting medium on Boehler–Almax anvils with 1000 µm culets and stainless-steel gaskets that were pre­indented to 78 and 67 µm. The samples were then compressed to 0.4 and 1.2 GPa, respectively.

**Figure 7:**
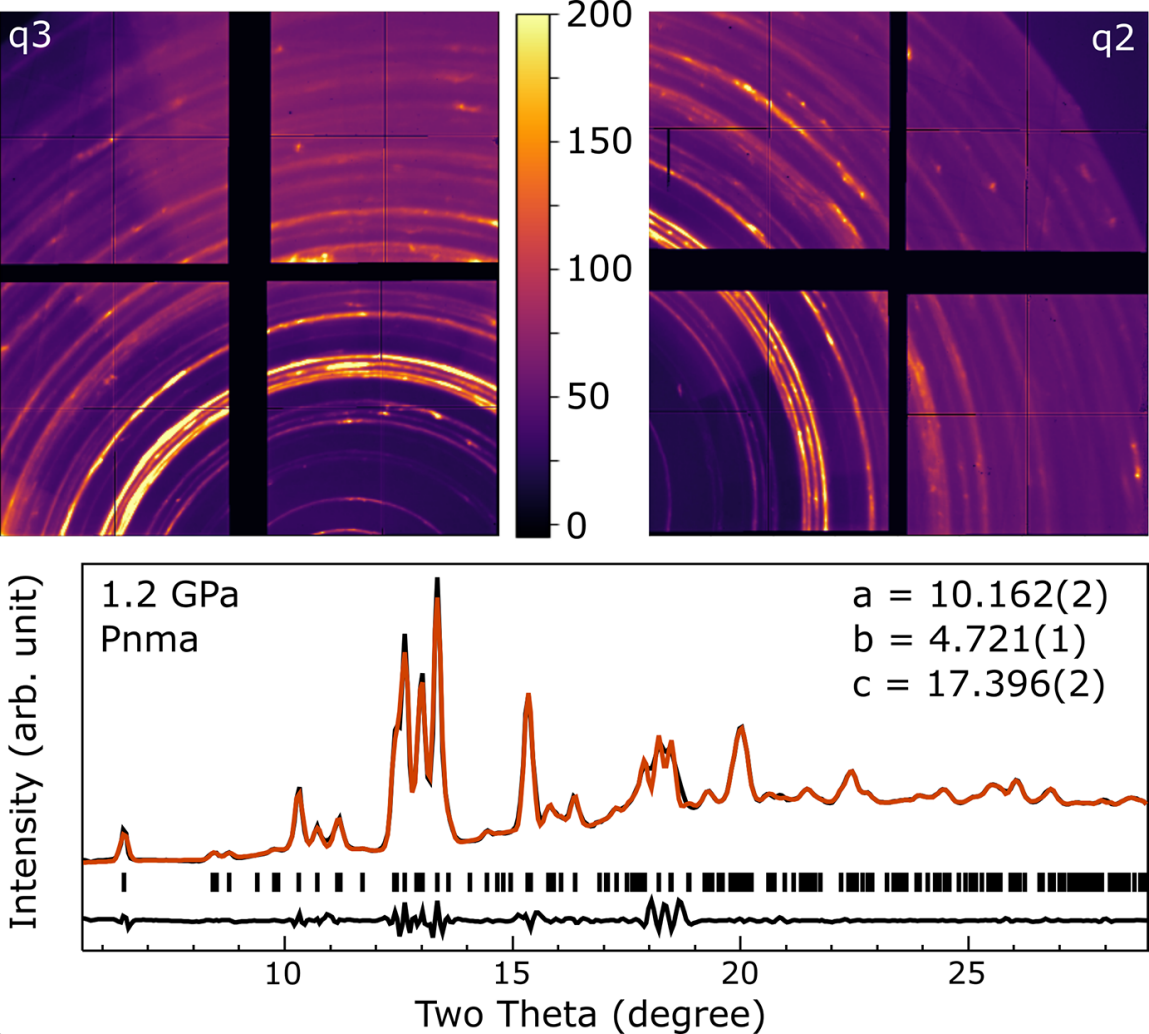
X-ray diffraction of the CsPbI_3_ sample at 1.2 GPa using 70% beam transmission. (Top) Mean of 500 pulses collected on each detector; the color scale is the same for both images. (Bottom) Integrated data from the patterns above combined into a single trace (black), shown with LeBail fit (orange) and residuals (lower black trace). Tick marks indicate the positions of allowed reflections. The fitted *Pnma* unit-cell parameters are *a* = 10.162 (2), *b* = 4.721 (1), and *c* = 17.396 (2) Å. No changes in the diffraction pattern were observed over the course of a run at 1.2 GPa.

Diffraction data are presented in Figs. 7 ▸ and 8 ▸. Fig. 7 ▸ shows data from the 1.2 GPa sample, which remained in the *Pnma* δ-phase and exhibited no phase transitions upon exposure to the XFEL beam.

**Figure 8:**
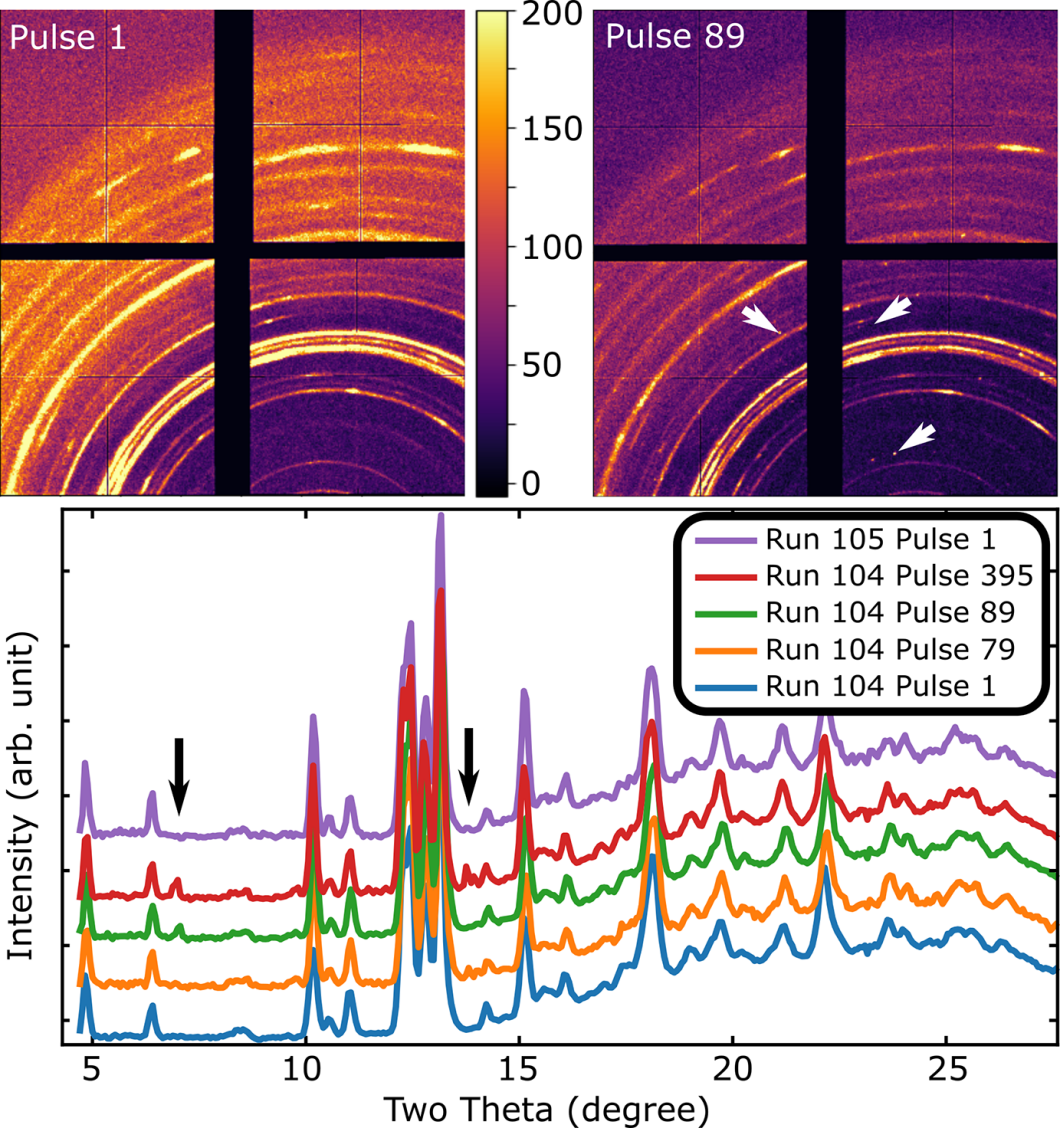
XFEL-induced transformation in CsPbI_3_ at 0.4 GPa during a run at 70% transmission with measured pulse energies of 149 (43) µJ. Note the emergence of new peaks during a run, marked by arrows. The sample has reverted to its initial state by the first pulse of the following run at the same location on the sample. The color scale is the same for both unintegrated patterns.

The sample at 0.4 GPa exhibits an XFEL-induced reversible phase transition upon exposure to the XFEL at beam transmissions of 70% and above. 50% transmission does not induce this transition. The transition is marked by additional peaks appearing in the diffraction pattern after ∼80 pulses. The representative evolution of the peaks during a run at 70% transmission is shown in Fig. 8 ▸. This is attributed to a transition to a per­ov­skite phase. The first new peak occurs at 2θ = 7.03°, which corresponds to a *d*-spacing of 6.09 Å. At ambient pressure, the α-phase of CsPbI_3_ has *a* = 6.290 Å at 593 K (Liu *et al.*, 2019 ▸), suggesting that this is the {100} peaks of the per­ov­skite. The next distinct new peak(s) (Fig. 8 ▸), observed at around 13.77°, likely corresponds to the {200} reflection family. This peak exhibits splitting, indicative of symmetry lowering of the per­ov­skite phase, and is best fit by the orthorhombic γ-phase, although the β-phase cannot be unambiguously excluded. Splitting of the {100} peak in either phase would be very slight and would not be resolved. Other peaks are overlapped by the complex diffraction from the residual *Pnma* phase (see Fig. 7 ▸) and are therefore unsuitable for fitting. Fits are presented in the supporting information.

The phase change is driven and sustained by irradiation at 120 Hz (8.3 ms between pulses), while at the beginning of the next run, approximately 1 min later, the CsPbI_3_ reverts to the initial δ-phase (Liu *et al.*, 2019 ▸). The thermal relaxation time of a DAC is much shorter than the 8.3 ms between pulses (Meza-Galvez *et al.*, 2020 ▸), implying that the high-temperature phase can be transiently quenched to ambient temperature. Whether this arises from metastability of the high-temperature per­ov­skite phase(s) or from modifications to the properties induced by the intense XFEL radiation, for example, the promotion of core electrons modifying the chemistry (Siska *et al.*, 2024 ▸; Konopkova *et al.*, 2025 ▸), is beyond the scope of this article. This serves as an example of long-timescale phase transitions at XFELs and presents an intriguing prospect for the continuous 1 MHz mode of the planned LCLS-II-HE upgrade, which is expected to deliver photons of high enough energies for DAC experiments (Raubenheimer, 2018 ▸) and could follow the evolution of a sample at 1 µs temporal resolution over any duration.

## 4. Comparison to existing facilities

DACs have previously been fielded on XFEL sources at EuXFEL and PAL-XFEL. Table 1 ▸ gives an overview of the capabilities of LCLS and EuXFEL which are pertinent to DAC experiments. Compared with the HED instrument at EuXFEL, the LCLS offers a different but complementary set of experimental capabilities, as outlined here. PAL-XFEL has a maximum photon energy of 20.6 keV, though previous DAC studies there have used much lower energy around 12 keV (Pace *et al.*, 2020 ▸). The XFEL operates at 60 Hz with greater than 10^12^ photons per pulse at 12.4 keV. Fewer DAC studies have been performed at PAL-XFEL than the dedicated DAC setup at the EuXFEL HED instrument, so the rest of the comparison focuses on that.

The pulse and photon energies of both LCLS and EuXFEL are comparable; however, the temporal structures of the pulses are quite different, and this determines the types of experiments to which each facility is best suited. The hard X-ray linac at LCLS operates at 120 Hz continuously. There are a wide variety of modes which can deliver 2 to 8 pulses each cycle with separations from a few femtoseconds to 120 ns depending on the method used (see Table 3 ▸). Currently available detectors can resolve each event at 120 Hz, but not individual pulses within, so the data in multiple pulse modes will be overlaid. It is possible to have differing photon energies between the pulses, so the absorption edge of foils can be used to attenuate one relative to the other on the detectors. More discussion of LCLS pulse modes is given in the *Outlook and future developments* section (Section 5).

EuXFEL operates in bursts of X-ray pulses spaced by integer multiples of 222 ns. Generally 2.2 or 4.5 MHz are used, though others, for example, 1.1 and 0.56 MHz, are also available, with bursts delivered at 10 Hz. The AGIPD detector can resolve up to 352 pulses at the burst frequency (Allahgholi *et al.*, 2015 ▸), so this many are generally used to cover 160 or 78.2 µs (for 2.2 and 4.5 MHz, respectively), depending on the burst frequency. This has proven to be a powerful tool for X-ray pump–probe experiments investigating phase transitions and chemical reactions on microsecond timescales, but it cannot resolve phenomena faster than the 222 ns period of the 4.5 MHz pulse trains. The two XFEL temporal structures are therefore complementary and their selection depends on the timescale of the phenomena of interest.

A final difference is in terms of configuration maturity. EuXFEL HED IC2 is optimized for DAC experiments and offers a streamlined user experience with a state-of-the-art streaked optical pyrometry system to measure temperature. Currently, there is no standard configuration for DAC studies at LCLS, and certain capabilities are yet to be commissioned. The final section of this article discusses future developments which would aid in fielding DACs at LCLS, along with some of the capabilities it could enable.

## 5. Outlook and future developments

A number of additional diagnostics can be added to improve the range of capabilities of DAC studies at LCLS. High-resolution online microscopy to view samples *in situ* would facilitate alignment of the XFEL on specific sample features. Pyrometry for temperature measurement would be valuable, and various fast pyrometers for DAC use have been reported recently (Ball *et al.*, 2023 ▸; Montgomery *et al.*, 2018 ▸). A gas-membrane system would allow the pressure to be adjusted *in situ* without breaking the chamber interlock. All of these features are currently available at other facilities and could be added as needed by users or as part of future upgrades to support DAC experiments at LCLS.

The LCLS beam itself is also subject to continuous up­grades, both in terms of the pulse modes available and the planned LCLS-II HE (high energy) upgrade to the superconducting linac. This will offer photon energies to at least 13 keV, and possibly as high as 20 keV, with a sustained repetition rate of 1 MHz. This would enable time-resolved studies of slower phenomena and could offer 1 µs sampling over any desired period. It will also offer extremely high average flux, so could enable photon-limited experiments, such as high resolution inelastic X-ray scattering.

In addition to more exotic pulse structures, several existing capabilities at LCLS could be applied to DAC experiments but were beyond the scope of this initial proof-of-concept study. For instance, optical laser-pump XFEL-probe studies of materials using femtosecond to nanosecond pulsed lasers are possible. MEC is equipped with an Nd:glass nanosecond-long pulse laser system with the pulse duration tunable from 2 to 35 ns. In a highly attenuated form, this could be used to transiently heat samples prior to the arrival of an XFEL probe pulse to investigate high-temperature phenomena.

Several LCLS instruments, including MEC, CXI, XCS, and XPP, have femtosecond short-pulse laser systems with various wavelengths, energies, and pulse durations, for use in laser-pump XFEL-probe studies. For short-pulsed lasers, the ablation threshold of a diamond surface is ∼2 J cm^−2^ (Komlenok *et al.*, 2011 ▸), which places limits on the pulse energy which can be delivered without damaging the anvils. The MEC short-pulse laser beam width is 65 mm, so using the standard ∼330 mm focal length off-axis parabolic focusing mirror and assuming an anvil thickness of 1.7 mm, the spot size on the diamond table would be ∼0.09 mm^2^, depending slightly on the actual anvil thickness and angle between the cell and laser. This would limit the pulse energy to 1.8 mJ. This gives a power density at the diamond-air surface of ∼5 × 10^13^ W cm^−2^, which is similar to the maximum possible before dialectic breakdown of air (Daigle *et al.*, 2010 ▸). This pulse energy is sufficient for many pump–probe experiments which could be performed at high pressure in DACs, for example, investigating optically switched electronic properties (Stojchevska *et al.*, 2011 ▸), transport properties (Lin & Zhigilei, 2007 ▸), and electron–phonon coupling (Mo *et al.*, 2024 ▸).

The DAC setup, as designed, is not tied to a single instrument at LCLS and could, in principle, be implemented at almost any instrument. This could enable a wide range of measurements at LCLS where the diamond anvils do not interfere, such as X-ray spectroscopy, small-angle X-ray scattering, X-ray imaging, and inelastic X-ray scattering.

## 6. Conclusion

In conclusion, we have demonstrated a flexible platform designed to field DACs at LCLS and presented data from various samples collected at the MEC instrument using the newly commissioned capability to run in air with a diamond window separating the chamber from the XFEL. The collection of high-quality powder X-ray diffraction data is demonstrated, paving the way for future DAC experiments at LCLS. Several novel experimental capabilities are suggested, as well as a complementary alignment procedure for DACs on XFEL sources utilizing a miniature YAG screen on the upstream anvil to image the XFEL beam.

## Supplementary Material

Supplemental figure supporting a phase assignment in CsPbI3. DOI: 10.1107/S1600577526001608/yi5188sup1.pdf

## Figures and Tables

**Table 1:** Comparison of selected DAC relevant parameters for LCLS and EuXFEL The LCLS has potential to probe short-time phenomena currently inaccessible at EuXFEL, which is better suited to longer timescale phenomena, such as chemical reactions and thermal conductivity. Note that the pulse energy is greatly decreased for both sources at the highest photon energies.

Parameter	LCLS	EuXFEL HED
Maximum photon energy	25 keV	25 keV
Pulse energy at 17 keV	∼1 mJ	∼1 mJ
Pulse delay	0 to 120 ns	Multiples of 222 ns
Repetition rate	120 Hz	Bursts of 4.5, 2.2, 1.1 and 0.56 MHz
Pulse duration	<50 fs	<50 fs
Polarization	Vertical	Horizontal

**Table 2:** X-ray parameters used

Parameter	Value
Photon energy	16.6 keV
Total pulse energy	0.6 mJ
Mode	Single pulse
Repetition rate	120 Hz
Pulse duration	<50 fs
Detectors	2× ePix10k

**Table 3:** DAC relevant LCLS multiple pulse modes

Mode	Pulse separation	Energy per pulse	Photon energy separation
Split undulator SASE (Lutman *et al.*, 2013 ▸)	0–30 fs	40 µJ	Up to factor of two
Two SASE pulses (Marinelli *et al.*, 2015 ▸)	0–125 fs	300 µJ	0.2–2%
Two bucket (Decker *et al.*, 2022 ▸)	Up to 120 ns in 350 ps increments	500–1000 µJ	∼1%
Multi-bucket (Decker *et al.*, 2022 ▸)	4 or 8 pulses at 700 ps	To be tested	–

**Table 4:** Overview of samples

Sample	Pressure (GPa)	Culet size (µm)
Cerium dioxide	4	1000
Cr + ammonia borane	26	400
CsPbI_3_	0.4	1000
CsPbI_3_	1.2	1000

## Data Availability

Data presented are available on request.
