# Supplementary material for: Demonstration of a diamond anvil cell platform at the Linac Coherent Light Source: capabilities and outlook
Source: J Synchrotron Radiat. 2026 Mar 26;33(Pt 3):552–61. doi: 10.1107/S1600577526001608 (PMC13148624; doi:10.1107/S1600577526001608)
Supplement: Supplementary file 1 [file s-33-00552-sup1.pdf]

JOURNAL OF SYNCHROTRON RADIATION

Volume 33 (2026)

Supporting information for article:

Demonstration of a diamond anvil cell platform at the Linac Coherent Light Source: capabilities and outlook

Mungo Frost, Nina Boiadjieva, Minkyung Han, Quynh L. Nguyen, Mengnan Wang, Hannah Bartels, Eric Galtier, Shao Xian Lee, Hemamala I. Karunadasa, Gilliss Dyer, Siegfried H. Glenzer, Wendy L. Mao, Yu Lin and Hae Ja Lee

Supplemental Material – Diamond anvil cell setup for X-ray free electron laser experiments at the Linac Coherent Light Source (LCLS)

Mungo Frost [a], Nina Boiadjieva [a], Minkyung Han [b], Quynh L. Nguyen [a], Mengnan Wang [b], Hannah Bartels [c], Eric Galtier [a], Shao Xian Lee [a], Hemamala I. Karunadasa [a, c], Gilliss Dyer [a], Siegfried H. Glenzer, [a], Wendy L. Mao [a, b], Yu Lin, [a], and Hae Ja Lee [a]

[a]SLAC National Accelerator Laboratory, 2575 Sand Hill Road, Menlo Park, CA 94025, USA
[b]Department of Earth and Planetary Sciences, Stanford University, Stanford, CA 94305, USA
[c]Department of Chemistry, Stanford University, Stanford, CA 94305, USA

S1 $CsPbI_3$ Phase Identification

Figure S1 shows the mean of 2586 diffraction patterns taken on $CsPbI_3$ at 0.4 GPa during a run at 80% transmission showing peaks from the perovskite phase which forms from the δ phase on exposure to the X-ray beam. Peak positions and intensities for the α, β and γ phases are shown along with those of the δ phase. Splitting of the higher angle perovskite peak implies distortion from the cubic α phase. While the β phase cannot be unambiguously ruled out, the orthorhombic γ phase has the best fit and is the most likely candidate.

The density is extrapolated to 0.4 GPa using the following bulk moduli: α: 15.23 GPa, β: 15.35 GPa, γ: 14.80 GPa, (Fadla *et al.*, 2020) and δ: 19.8 GPa (Rakita *et al.*, 2015).

References

Fadla, M. A., Bentria, B., Dahame, T. & Benghia, A. (2020). *Physica B: Condensed Matter*, **585**, 412118.

Rakita, Y., Cohen, S. R., Kedem, N. K., Hodes, G. & Cahen, D. (2015). *Mrs Communications*, **5**(4), 623–629.

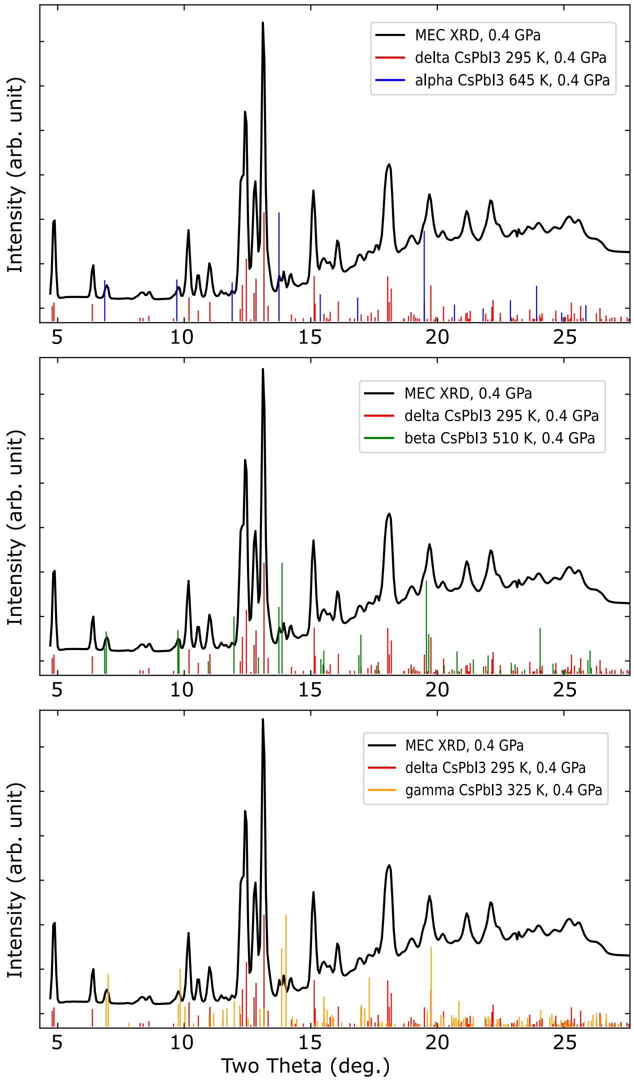

Figure S1: Pattern of $CsPbI_3$ at 0.4 GPa in a run with 80% transmission. Data in black with calculated positions and intensities for different phases shown below. The starting δ-phase is shown in red for all panels. **Top:** The α-phase (blue) does not exhibit splitting near 13.8° and is an unlikely candidate. **Middle:** The β-phase (green) has the peaks near 13.8° split but does not have weak peaks at 11.4 to 11.7°. **Bottom:** The γ-phase has both splitting and intensity near 11.5° and is the most likely candidate.
